# Interferon-α inducible protein 6 impairs EGFR activation by CD81 and inhibits hepatitis C virus infection

**DOI:** 10.1038/srep09012

**Published:** 2015-03-11

**Authors:** Keith Meyer, Young-Chan Kwon, Shuanghu Liu, Curt H. Hagedorn, Ratna B. Ray, Ranjit Ray

**Affiliations:** 1 Department of Internal Medicine, Saint Louis University; 2 Department of Medicinal Chemistry, College of Pharmacy, University of Utah; 3 Department of Medicine and Genetics, University of Arkansas for Medical Sciences; 4 The Central Arkansas Veterans Healthcare System; 5 Department of Pathology, Saint Louis University; 6 Department of Molecular Microbiology & Immunology, Saint Louis University

## Abstract

Viral entry requires co-operative interactions of several host cell factors. Interferon (IFN) and the IFN-stimulated genes (ISGs) play a central role in antiviral responses against hepatitis C virus (HCV) infection. We examined the effect of interferon-α inducible protein 6 (IFI6) against HCV infection in human hepatoma cells. HCV RNA level or infectious foci were inhibited significantly by ectopic expression of IFI6. IFI6 impaired CD81 co-localization with claudin-1 (CLDN1) upon HCV infection or CD81 cross-linking by specific antibody. Activation of epidermal growth factor receptor (EGFR), a co-factor involved in CD81/CLDN1 interactions, was reduced in IFI6 expressing cells in response to HCV infection or CD81 cross linking by antibody, but not by treatment with EGF. Taken together, the results from our study support a model where IFI6 inhibits HCV entry by impairing EGFR mediated CD81/CLDN1 interactions. This may be relevant to other virus entry processes employing EGFR.

HCV is a major cause of chronic hepatitis worldwide; often leading to chronic liver disease with the potential for development of hepatocellular carcinoma. The essential and conserved nature of the entry step in the HCV life cycle offers an attractive target for therapeutic intervention. HCV entry appears to be a complex multistep process involving viral envelope glycoproteins as well as several cellular attachment and entry factors^1^,^2^,^3^,^4^,^5^. HCV envelope glycoprotein E2 binds human CD81, a tetraspanin expressed on many cell types including hepatocytes and B lymphocytes^1^. Tetraspanins are thought to exert their biological function(s) by co-ordinating the trafficking of associated ligands into tetraspanin-enriched microdomains. A second transmembrane domain protein family, the Claudin superfamily, is the major structural component of cellular tight junctions and associated with HCV entry^3^,^6^,^7^. Antibodies that inhibit HCV interactions with CD81 neutralize multiple genotypes of HCV^8^. Modulation of HCV specific entry factor interactions has been observed to reduce HCV viral titers in cell culture^9^,^10^. Inhibition of host cell kinase function inhibits HCV replication after binding of the virus to the cell surface, with a loss of CD81 translocation to the tight junctions in a post binding step^6^. Although HCV is known to enter hepatocytes via clathrin-mediated endocytosis^11^, the host-virus interactions governing HCV internalization are not well understood. HCV was recently demonstrated to induce CD81 and claudin 1 (CLDN1) endocytosis^7^, although the molecular interactions important for HCV internalization still remain unclear.

HCV entry is a multistep process and reported to be regulated by a 170 kDa EGFR protein^12^. EGFR is a member of a family of receptor tyrosine kinases which lies at the head of a complex signal transduction cascade that modulates cell proliferation, survival, adhesion, migration and differentiation. EGFR consists of an extracellular ligand-binding domain, a transmembrane lipophilic domain, an intracellular tyrosine kinase domain and the C-terminal region with multiple tyrosine residues^13^. Ligand binding to the EGFR results in receptor dimerization, activation of an intracellular kinase domain, autophosphorylation of tyrosine residues, internalization, and lysosomal degradation^14^,^15^. These phosphorylated tyrosine residues recruit and activate downstream signaling pathways including the Ras/Raf/mitogen-activated protein kinase (MAPK) pathway, extracellular signal-regulated kinase (ERK), signal transduction and activator of transcription (STAT), and a serine/threonine (AKT) signaling machinery.

IFNs are a well-known family of cytokines with antiviral effects^16^,^17^. IFNs modulate cellular proliferation and stimulate immune responses through ISGs. IFI6 is a type I ISG^18^,^19^, and plays a critical role in regulating apoptosis. Increased expression of IFI6 has been associated with the anti-apoptotic activity of IFN-α2b. IFI6 is a mitochondria-targeted protein, inhibits the release of cytochrome c from mitochondria and delays the apoptotic process initiated and transduced by the TNF-related apoptosis-inducing ligand/caspase 8 pathway^20^. Conversely, RNA interference-mediated down regulation of IFI6 restored IFN-α2b-induced apoptosis. IFI6 is strongly associated with the immune system, but its antiviral effects are not well known^21^. IFI6 and IFI27 are two related proteins belonging to the FAM14 family on the basis of sequence similarity that are commonly induced by IFNs. Emerging studies of IFI6 and IFI27 suggest that both are mitochondrial proteins with opposing activities on apoptosis that may regulate innate immune responses of IFNs. IFI27 is strongly induced by IFN-α and to a lesser extent by IFN-γ in several cell lines^22^. IFI6 expression decreases yellow fever virus (YFV) titer in cell culture^23^. IFI6 strongly regulates Dengue 2 virus^24^ and West Nile virus (WNV) infection^25^. We have previously identified a number of ISGs which were modulated in HCV infected cell culture, and infected cells exposed to interferon^26^. The expression of IFI6 and IFI27 were significantly reduced after HCV infection of interferon pre-treated cells as compared to mock infected IFN pre-treated Huh7.5 cells.

Receptor kinase function has been implicated in HCV infection. The binding of HCV particles to human hepatocytes induces EGFR activation, which is dependent upon interactions with CD81^12^. EGFR can also be activated by antibody mediated cross-linking of CD81^27^. EGFR is important in the entry process for multiple viruses, and recognized as a host cofactor for HCV entry^12^. Blocking EGFR kinase activity impaired infection of all major HCV genotypes in cell culture, and in a human liver chimeric mouse model. Here, we have ectopically expressed IFI6 in Huh7.5 cells to examine the effect on HCV infection in the absence of an apparent IFN response. We report that exogenous expression of IFI6 limited HCV entry and replication. We have identified IFI6 as directly modulating HCV infection through CD81 interactions with CLDN1, via inhibition of EGFR kinase function in a signaling pathway specific manner.

## Results

### Inhibition of HCV replication by exogenous expression of IFI6 in Huh7.5 human hepatoma cells

A pathway-specific microarray was performed to obtain a comprehensive view of the changes in IFN signaling after HCV infection^24^. HCV infection of IHH modestly upregulated or inhibited the expression of IFN signaling molecules. On the other hand, the majority of the genes were upregulated in cells treated with IFN-α compared to levels for mock-treated cells. Interestingly, most of the ISGs were down regulated. Interestingly, the expression of several ISGs with known antiviral function was decreased as observed in our pathway-specific array when IFN-pretreated cells were infected with HCV. Among the genes regulated in this HCV infection model, we focused on IFI6 and IFI27; two members of the FAM14 family with identified ISG function, in this study to evaluate their potential role in modulating HCV infection.

**Figure 1:**
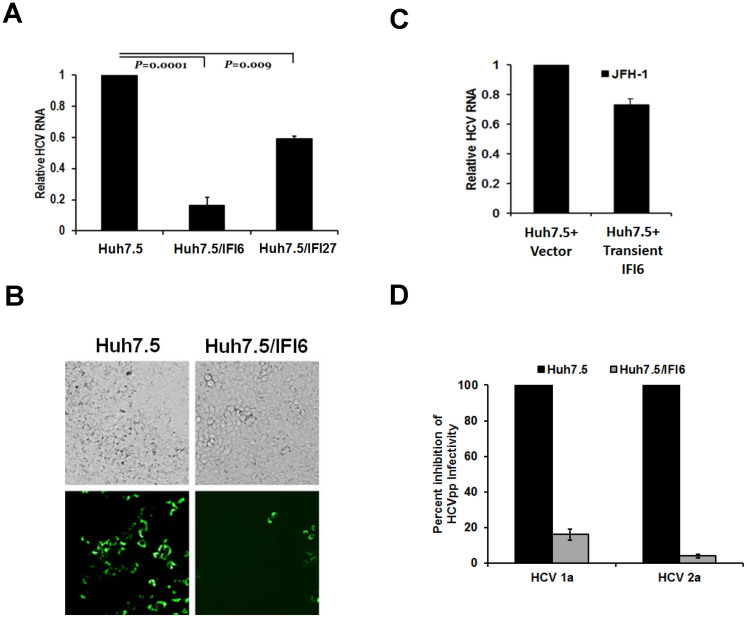
Exogenous expression of IFI6 and IFI27 inhibit E2 mediated cell entry of HCV. Huh7.5 cells stably expressing IFI6 or IFI27 were infected with HCVcc (m.o.i = 1) for 5 h at 37°C. Cells were rinsed and incubated for 96 hours at 37°C. Total RNA isolated from HCV infected cells was used for cDNA generation and quantification of viral RNA titers by real-time PCR. A standard curve of virus dilutions was used as a comparative control and 18S RNA as an internal control (panel A). HCV JFH1-EGFP was used to infect control and stably IFI6 expressing cells. Cell foci were counted after 48 hours by fluorescence (panel B). Transient transfection and expression of IFI6 in Huh7.5 cells produced a modest but specific reduction of HCV RNA levels (panel C). HCVpp pseudotype displayed a decreased viral infection as compared to positive controls in stably IFI6 expressing Huh7.5 cells (panel D). A minimum of three replicates for each set of experiments were done to compile the data as presented. Standard deviations are shown as error bars, wherever applicable.

To examine the effect of HCV infection upon selected ISGs, we infected IFI6 expressing Huh7.5 cells as well as vector transfected control cells with cell culture produced HCV genotype 2a (clone JFH1). A distinct level of HCV RNA inhibition was observed in IFI6 expressing cells (80–90%) or IFI27 cells (~40%) as compared to vector transfected control cells (Fig. 1, panel A). A significant reduction (80%) in the number of HCV EGFP foci was also observed in cells expressing IFI6 following infection with ~1,000 ffu of the virus (Figure 1, panel B). In IFI6 expressing cells there were significantly fewer infected foci; as visualized by GFP imaging, indicative of a reduced efficiency in HCV infection. Stable expression of exogenous proteins may cause unintended alterations of cell physiology, resulting in unintentional side effects. To verify that IFI6 specifically reduces HCVcc infection during transient expression, IFI6 was expressed in Huh7.5 cells 24 h prior to infection. As above, a specific reduction (~30%) in HCV RNA levels was observed in IFI6 expressing cells (Figure 1, panel C), indicating that the reduction of HCV was not due to aberrant effects during stable ISG expression. To test the possibility that IFI6 had a direct effect on HCV replication, IFI6 was transiently expressed in Huh7.5 cells harboring the complete HCV replicon (Rep2a, HCV subtype 2a). HCV RNA levels were only minimally decreased (<15%) by the expression of IFI6, as compared to controls (Figure S1 in File S1, panel A). Furthermore, expression of HCV NS3 protein was minimally decreased (<20%) during transient expression of IFI6 (Figure S1 in File S1, panel B).

To further characterize the IFI6 mediated inhibition of HCV infection, we determined if IFI6 some way modified cell entry receptors (Figure 1, panel B). We used a pseudotype virus to separate effects of IFI6 on HCV receptor interactions from viral replication. Cell lines stably expressing IFI6 were used for these experiments. Vector transfected cells were used in parallel as negative controls. HCVpp pseudotype virus, expressing both E1 and E2 glycoproteins from either the genotype 1a or 2a on the external surface, exhibited a decrease in RLU in cells expressing IFI6 protein as compared to control cells (Figure 1, panel D), suggesting that the expression of IFI6 had an inhibitory effect at an early step of HCV infection.

### IFI6 localizes on the surface and intracellularly in Huh7.5 cells and does not associate with CD81

**Figure 2:**
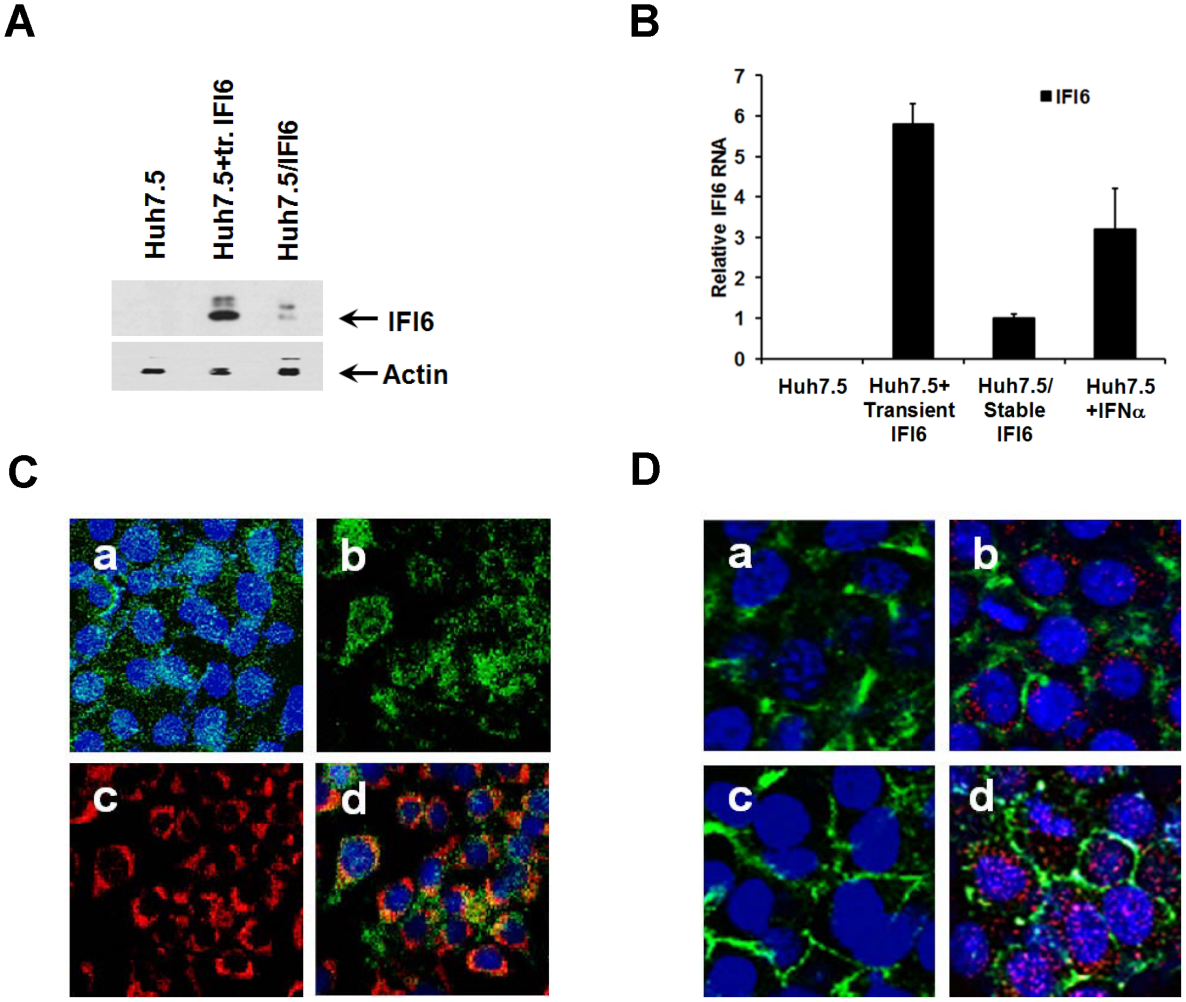
IFI6 localizes on both the cell surface and intracellularly, and does not associate with CD81. FLAG tagged IFI6 expression was detected in lysates of Huh7.5 stable transfectants by Western blotting with a FLAG specific antibody. Note that cropped gel images are used in this figure and the gels were run under the same experimental conditions (panel A). Expression of IFI6 is shown during transient, stable, and IFN-α stimulated conditions; and standard deviations are shown as error bars (panel B). Detection of IFI6 on the cell surface of unfixed (panel C, section a) and in the cytoplasm of fixed cells (panel C, section b) by FLAG antibody. Mitochondria were stained with a mitotracker dye (panel C, section c) and merged fluorescence of cytoplasmic IFI6 and stained mitochondria (panel C, section d) are shown. Basal expression of CD81 was determined in Huh7.5 cells (panel D, Section a) and IFI6 transfected Huh7.5 cells (panel D, section b). Huh7.5 and IFI6 expressing cells were cross-linked with a CD81 specific antibody, followed by fixation. CD81 translocation after antibody mediated cross-linking was observed in control (panel D, section c) and was not inhibited in IFI6 expressing cells (panel D, section d). The presence of IFI6 (red) was also observed (panel C, section D) and did not co-localize with CD81 (green). Cell nuclei were stained with DAPI (blue). Each panel is representative of three independent experiments.

Huh7.5 cells were transiently or stably transfected with IFI6 plasmid DNA (Huh7.5/IFI6). Rep2a cells were also transiently transfected to express IFI6. Transient and stable expression of IFI6 in Huh7.5 cells was documented by Western blot analysis using antibody to FLAG, as compared to controls (Fig. 2, panel A). Relative levels of IFI6 mRNA were measured by real time PCR following stable and transient expression. IFI6 expression in the presence of IFN-α was examined as a positive control. Expression of IFI6 was detected in unstimulated transiently transfected Huh7.5 cells. Expression of IFI6 at the RNA level in stable transfectants was modest and was approximately six fold less than that seen during transient transfection, and three fold less than that seen in an IFN-α treated cell line (Figure 2, panel B). As noted previously (Fig. 1, panels A and C) there is a decreased ability of IFI6 to reduce HCVcc infection in transiently transfected cells, and this difference is likely due to the reduced number of cells expressing IFI6 in the total cell population. Immunofluorescence staining of IFI6-FLAG expressing cells exhibited protein localization as punctate dots on the surface of the unfixed cells (Fig. 2, panel C, section a), as well as a more localized expression upon intracellular staining (Fig. 2, panel C, section b). Mitotracker Red was used to stain mitochondria (Fig. 2, panel C, section c). Intracellular staining of IFI6 was done in fixed cells and determined that there was significant co-localization of IFI6 with mitochondria (28%) using a mitotracker stain (Fig. 2, panel C, section d).

To determine whether structural proteins of HCV may have an association with IFI6, 293 cells were transfected with plasmid constructs for expressing IFI6-FLAG, and HCV Core, E1 or E2. Transfected cells were examined for both the co-localization, and co-immunoprecipitation of these proteins under low stringency conditions. There was no co-localization or co-immunoprecipitation observed between IFI6 and HCV structural proteins (data not shown). Earlier experiments conducted by others did not observe co-immunoprecipitation of non-structural HCV proteins and IFI6^28^. Together, these results indicated that there were no detectable direct interactions between the IFI6 and E1 or E2 proteins that might modulate virus infection.

CD81 expression was examined in control Huh7.5 cells and IFI6 expressing Huh7.5 cells (Fig. 2, panel D). Cells expressing IFI6 exhibited diffuse localization of CD81 on the cell surface (Fig. 2, panel D, section b), similar to control cells (Fig. 2, panel D, section a). Cross-linking of CD81 with JS81 antibody in control cells led to the translocation of CD81 to areas associated with tight junction formation (Figure 2, panel D section c). Another ISG, IFITM1, localizes to tight junctions after interferon stimulation and interacts with HCV co-receptors CD81 and occludin to inhibit HCV entry^29^. However, IFI6 did not localize to areas of tight junction formation or CD81 after antibody crosslinking of CD81 (0.8% co-localization of IFI6 with CD81 per field), and did not affect CD81 translocation (Fig. 2, panel D, section d). Thus, IFI6 had no detectable effect on CD81 localization after stimulation (by cross-linking) as compared to controls. Possible interactions between IFI6 and CD81 were further evaluated by co-immunoprecipitation assays under low stringency conditions and results showed no evidence for co-precipitation using antibodies to either CD81 or IFI6-FLAG (data not shown). These results indicate that there is no detectable direct association between CD81 and IFI6, and that IFI6 does not directly affect the movement of CD81 on the cell surface.

### Exogeneous expression of IFI6 in Huh7.5 cells inhibits CD81 co-localization with CLDN1

**Figure 3:**
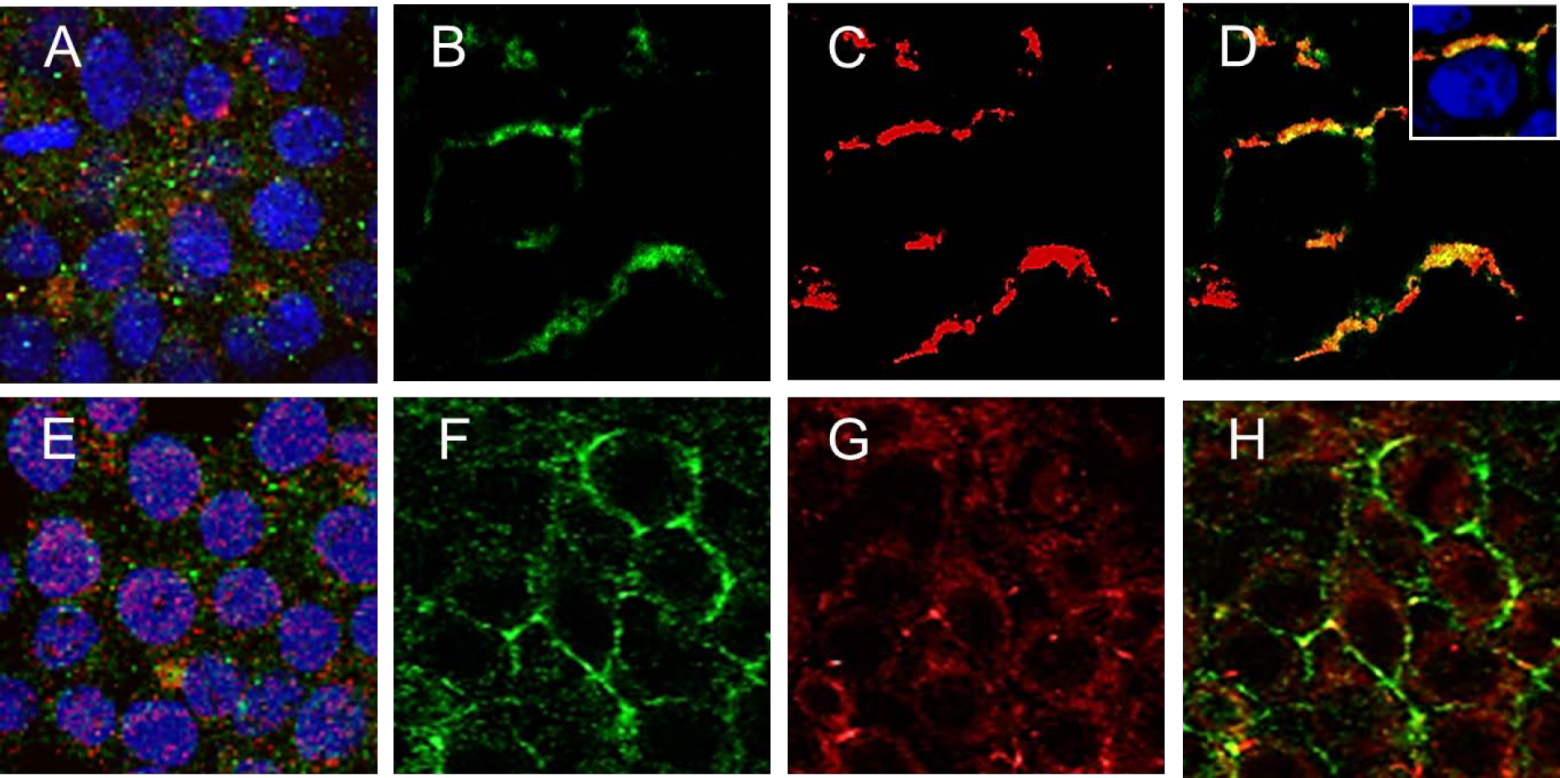
IFI6 expression inhibits CD81 and CLDN1 interactions during antibody cross-linked activation of CD81. Expression of CD81 and CLDN1 on the cell surface of unstimulated control and Huh7.5 stable transfectants expressing IFI6 is shown (panels A and E; respectively). CD81 (panel B) and CLDN1 (panel C) were detected in fixed mock-transfected control Huh7.5 cells. Merged fluorescence showing translocation of CD81 and co-localization with CLDN1 in tight junctions of cells (panel D, 56% co-localization of claudin with CD81 per field). CD81 (panel F), CLDN1 (panel G), and merged immunofluorescence analysis displaying localization (panel H, <0.1% co-localization of claudin with CD81 per field) are shown in IFI6 transfected cells. Each panel is representative of three independent experiments.

The possibility that HCV entry dependent on co-receptor complex formation between the tetraspanin superfamily member CD81 and the tight junction protein claudin1 (CLDN1) on cell membranes was tested. Control and IFI6 expressing Huh7.5 cells were incubated with CD81 specific JS-81 antibody for 60 minutes at 37°C, followed by fixation and permeabilization^11^,^30^. Co-localization of CD81 and CLDN1 proteins was observed in control cells (8.9% co-localization of CD81 with CLDN1 per field, 56% co-localization in selected stimulated cells) (Fig. 3, panels B-D), while limited co-localization of CD81 and CLDN1 was observed in cells expressing IFI6 protein as measured by immunofluorescence (<0.1% co-localization of CD81 with CLDN1 per field) (Fig. 3, panels F-H). Unless indicated, co-localization was measured in triplicate using confocal images taken at 20x of no fewer than 100 cells. These results suggested that HCV entry might be impaired due to a lack of CLDN1 and CD81 co-receptor interaction in cells expressing IFI6.

**Figure 4:**
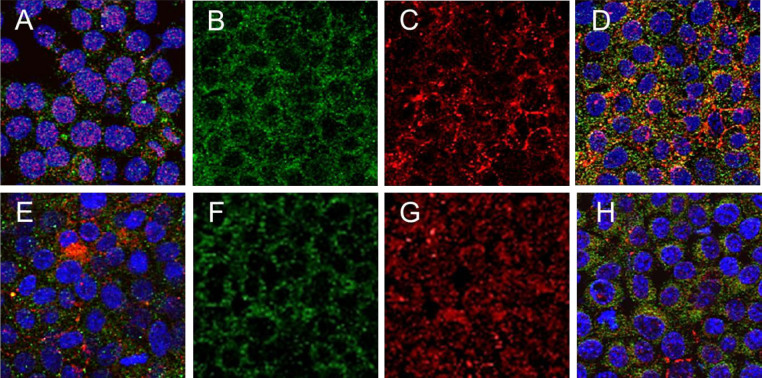
IFI6 expression inhibits CD81 and CLDN1 interactions during HCV infection. Huh7.5 control and IFI6 expressing cells were infected with HCVcc and co-localization of CD81 and CLDN1 was determined. Expression of CD81 and CLDN1 on the cell surface of unstimulated control and IFI6 expressing cells is shown (panel A and E, respectively). CD81 (panel B) and CLDN1 (panel C) were detected in fixed mock-transfected control cells. Merged immunofluorescence showing translocation of CD81 and co-localization with CLDN1 in tight junctions of cells is shown (panel D, 6.1% co-localization of CLDN1 with CD81 per field). CD81 (panel F), CLDN1 (panel G), and merged fluorescence displaying localization (panel H, 0.2% co-localization of CLDN1 with CD81 per field) are shown in IFI6 transfected cells. Each panel is representative of three independent experiments.

CLDN1 and CD81 co-localize on the surface of Huh7.5 cells shortly after inoculation with HCVcc^11^,^30^. Huh7.5 cells expressing IFI6 were exposed to HCVcc (clone JFH1) for 2 hours at 4°C, followed by an additional incubation of 1 hour at 37°C. CLDN1 and CD81 co-localized in control Huh7.5 cells (6.1% co-localization of CD81 with CLDN1 per field) (Figure 4, panels B-D). In contrast, a significant reduction in CD81 and CLDN1 co-localization was analyzed in cells expressing IFI6 (0.2% co-localization of CD81 per field) (Figure 4, panels F-H). These results provide evidence that IFI6 inhibits the co-localization of CLDN1 and CD81 shortly after exposure to HCV and suggests that inhibitory effect of IFI6 on HCV infection is an early event, such as cell entry.

### CD81 cross-linking reduces activation of EGFR in cells expressing IFI6

EGFR belongs to the erythroblastic leukemia viral oncogene homolog (ERBB) family of receptor tyrosine kinases. Ligand-induced receptor dimerization and autophosphorylation of distinct tyrosine residues of EGFR creates docking sites for specific membrane-targeted proteins. Distinct downstream signaling cascades are initiated by EGFR depending on its phosphorylation pattern. One of the predominant C-terminal phosphorylation sites of EGFR is Tyr1068, which is used to analyze ligand-induced activation of EGFR and is associated with downstream signaling following ligand binding to CD81^12^,^27^. The receptor tyrosine kinase EGFR acts as a cofactor for HCV entry by promoting CD81-CLDN1 complex formation through the HRas mediated lateral movement of CD81^6^.

**Figure 5:**
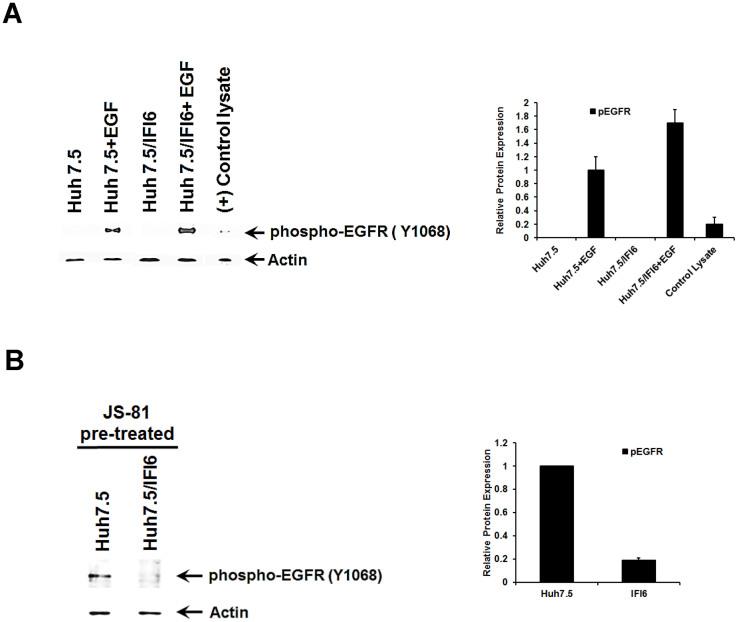
IFI6 expression inhibits EGFR activation in the presence of CD81 antibody. IFI6 stably transfected cells were treated with EGF and cell lysates were analyzed for phosphorylated EGFR. Expression of IFI6 inhibited EGFR activation by CD81 cross-linking, but not from EGF treatment (panel A). Cells stimulated with antibody to CD81 were lysed in a reducing sample buffer and subjected to Western blot analysis for EGFR activation (panel B). Blots were reprobed with antibody to actin as a loading control. Quantification of protein levels was performed using ImageJ software from three separate experiments and standard deviations are shown as error bars. Note that cropped gel images are used in this figure and the gels were run under the same experimental conditions.

The binding of HCV particles to human hepatocytes induces EGFR activation, which is dependent on interactions with CD81^11^, a process which may be replicated by antibody mediated cross-linking of CD81^27^. HCV entry requires HRas activation downstream of receptor tyrosine kinase EGFR signaling. EGFR ligands that enhance the kinetics of HCV entry induce EGFR internalization and co-localization with CD81, while EGFR kinase inhibitors inhibit HCV infection primarily by preventing EGFR endocytosis. Antibodies that block EGFR ligand binding, or inhibitors of EGFR downstream signaling, have no effect on HCV entry. To test if EGFR activation was inhibited in IFI6 expression, control and cells expressing IFI6 were treated with epidermal growth factor (EGF). EGF stimulated phosphorylation of EGFR to an equal extent in both control and IFI6 expressing cells (Figure 5, panel A), while it was not observed in untreated control or IFI6 expressing cells. Cross-linking of CD81 by JS81 antibody or HCVcc activates EGFR prior to movement to tight junctions^9^,^12^. Antibody cross-linking of CD81 in Huh7.5 control cells resulted in phosphorylation of EGFR at Tyr1068 (Figure 5, panel B). However, phosphorylation at Tyr 1068 was decreased (~19% of controls) in cells expressing IFI6 (Figure 5, panel B), as compared to controls. These results show that EGF is able to stimulate EGFR at Tyr1068 in IFI6 expressing cells in a similar manner to control cells, and that the inhibition of EGFR phosphorylation in an IFI6 expressing cells is confined to those interactions involving CD81.

### IFI6 expression inhibits the Ras/Raf1/Erk signaling pathway

**Figure 6:**
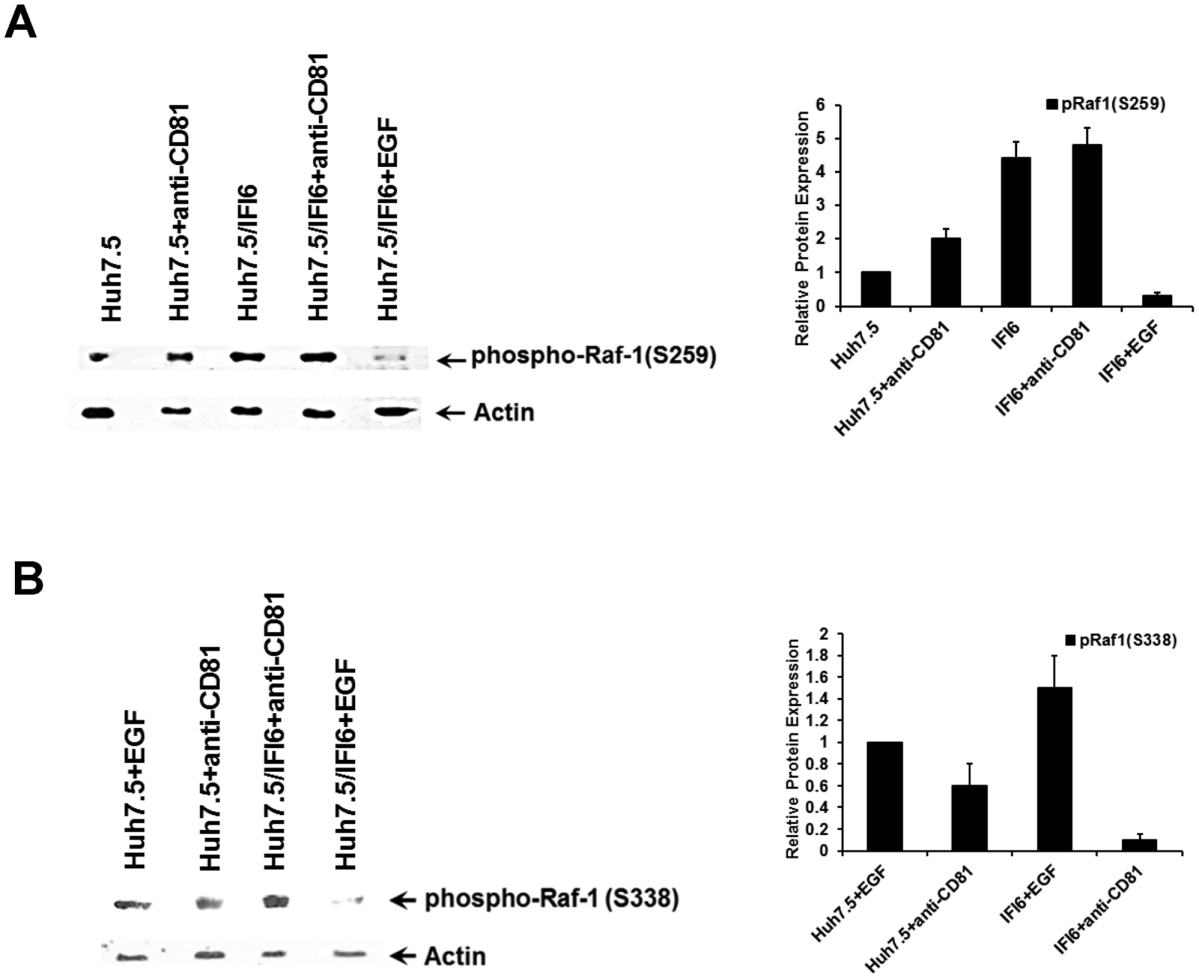
Activation of Ras/Raf/Erk pathway is inhibited in IFI6 expressing cells. Huh7.5 cells treated with antibody to CD81 for the indicated time were lysed in a reducing sample buffer and subjected to Western blot analysis. Lysates were probed for phosphorylated Raf-1 (Ser259). In addition, IFI6 expressing cells were stimulated with EGF and compared to antibody CD81 cross-linked cells to detect the loss of phosphorylation of Raf-1 at Ser259 (panel A). Activation of Raf-1 was analyzed by measuring phosphorylation at Ser338 cross-linking of CD81 or treatment with EGF (panel B). Blots were reprobed with antibody to actin as a loading control. Quantification of protein levels was performed using ImageJ software from three separate experiments and standard deviations are shown as error bars. Note that cropped gel images are used in this figure and the gels were run under the same experimental conditions.

Activated HRas regulates cell proliferation, migration, and invasion via the downstream effector-signaling pathways, Raf-1/MAPK/Erk and PI3K/Akt^31^,^32^. Raf-1 kinase is an important intermediate in the transduction of proliferative and anti-apoptotic signals. HRas binds with high affinity to Raf-1^33^ and recruits Raf-1 to the plasma membrane, where it is activated^34^,^35^. In the resting state, Ser259 of Raf-1 is phosphorylated and bound by 14-3-3, thus keeping it inactive by preventing the spontaneous association of Raf-1 with Ras-GTP. Raf-1 phosphorylation at Ser259 is significantly elevated in IFI6 expressing cells in comparison to unstimulated controls (Figure 6, panel A). Phosphorylation of Ser259 of Raf-1 is increased modestly in response to CD81 cross-linking in IFI6 expressing cells, as compared to control cells (Fig. 6, panel A). After cross-linking with a CD81 specific antibody, phosphorylation of Raf1 at Ser259 was maintained at a level twice that of Huh7.5 control cells. EGF treatment of cells leads to dephosphorylation of Raf-1 at Ser259^36^. We observed that the ability of EGF to dephosphorylate Raf-1 at Ser259 in IFI6 expressing cells was maintained (Fig. 6, panel A), indicating that inhibition of the upstream Ras/Raf/Erk pathway in cells expressing IFI6 was specific to the CD81 mediated induction of Raf-1.

**Figure 7:**
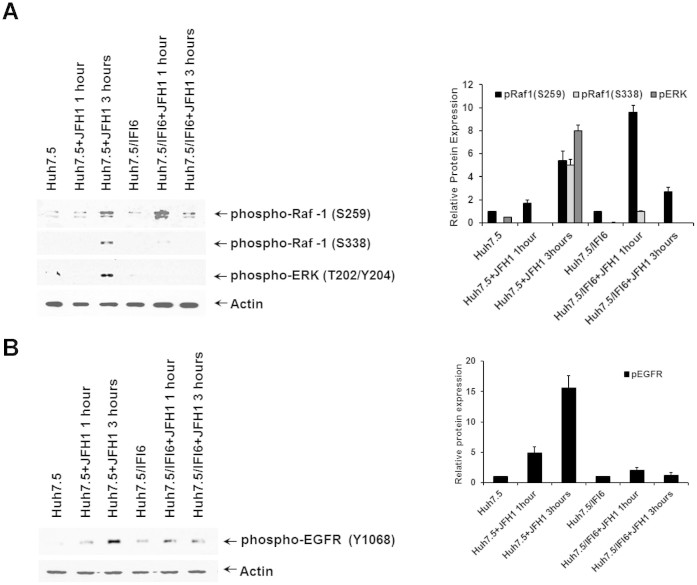
HCV infection of IFI6 expressing cells reduced activation of EGFR and the Ras/Raf/ERK pathway. IFI6 expressing and control Huh7.5 cells were infected with cell culture produced HCV (clone JFH1). Cellular responses to IFI6 expression in the Ras/Raf/ERK pathway (panel A), and the activation of EGFR (panel B) are shown. Quantification of protein levels was performed using ImageJ software from three separate experiments and standard deviations are shown as error bars. Note that cropped gel images are used in this figure and the gels were run under the same experimental conditions.

Ser338 of Raf-1 is autophosphorylated in response to EGF, and Raf-1 dimerization is required for Ser338 phosphorylation which is critical for the activation of Raf-1^37^. Ser338 phosphorylated Raf1 were not observed by Western blot analysis in cells not exposed to EGF, CD81 or HCVcc (Figure 7, panel A). After stimulation with either EGF or antibody to CD81 (JS81), we analyzed the ability of these ligands to stimulated phosphorylation of Raf at Ser338. Both IFI6 expressing and control cells showed similar levels of Raf1 phosphorylation at Ser338 following treatment with EGF. However, stimulation of the cells by cross-linked CD81 only induced phosphorylation of Raf1 at Ser338 in control cells, while IFI6 expressing cells showed limited (~6 fold less than controls) phosphorylation at Ser338 (15 fold less cells treated with EGF) (Fig. 6, panel B). Interestingly, activation of Erk following CD81 cross-linking was not compromised despite a lack of Raf-1 activation in IFI6 expressing cells (data not shown). Inhibition of Erk during HCV infection was reported not to cause a decrease in viral replication^6^, and modulation of virus infection was only observed through the inhibition of MAPK signaling (Raf and HRas) at sites close to EGFR.

### IFI6 expression decreases the activation of EGFR by HCV infection

Huh7.5 cells expressing IFI6 were inoculated with HCV genotype 2a (JFH1) for 1 and 3 hours prior to analyses for early cellular responses to virus infection. Stimulation of IFI6 expressing cells by incubation with HCVcc for 1 hour at 37°C resulted in increased phosphorylation of Ser259 in Raf-1 (Figure 7, panel A). Furthermore, activation of Raf-1 by HCVcc, as measured by phosphorylation at Ser338, was greatly reduced in IFI6 expressing cells (Fig. 7, panel A) suggesting that HCVcc mediated signaling via CD81 interactions are inhibited by IFI6. In contrast, strong activation of Raf-1 at Ser338 was observed in control cells. Interestingly, the activation of ERK under these conditions was different from that seen in response to CD81 cross-linking by JS81 antibody. Phosphorylation of ERK was not present in the IFI6 expressing cell line and only detected in control cells. This finding highlights a difference in HCV mediated signaling through ERK that is separate from its interactions with CD81 at the cell surface.

During the initial stages of HCV infection, the expression of IFI6 led to a modulated response in EGF receptor activation. Three hours post inoculation, HCV induced significant phosphorylation of Tyr1068 indicating activation of the receptor and stimulation of downstream tyrosine kinase activity (Fig. 7, panel B). In contrast, expression of IFI6 modulated the phosphorylation of Tyr1068, indicating that EGFR activation after HCV infection is reduced in IFI6 expressing cells.

## Discussion

The EGFR and downstream pathways play a critical role in cell entry of many viruses and is a host cofactor for HCV entry. Blocking EGFR receptor kinase activity impairs infection of all major HCV genotypes in cell culture, and in a human liver chimeric mouse model^17^. Our report provides evidence that IFI6 inhibits HCV infection by impairing CD81 and CLDN1 interactions by inhibiting the function of the EGFR kinase.

IFI6 was one of a number of ISGs shown to inhibit yellow fever virus (YFV) infection in STAT1-deficient human (STAT1^−/−^) fibroblasts or in human hepatoma cells^23^. Cell lines stably expressing IFI6 were infected with DENV-Fluc (NS4B:L52F), and were monitored from 12 to 72 hours. Cells expressing IFI6 were indistinguishable from control cells at the 12 hour time point indicating a lack of inhibition at receptor interaction or primary translation, but conferred inhibition at 24 hours and later time points, suggesting a block at a subsequent step in DENV replication^25^. Expression of IF6 does not significantly inhibit HCV RNA replication, but enhance the antiviral effect of IFN-α^18^. A previous study described the reduction in HCV RNA and core protein release in IFI6 expressing cells, but was unable to identify what step of viral infection was affected^28^. In this report we provide evidence that IFI6 inhibits HCV entry into cells suing HCV pseudotype particles and an infectious HCV JFH1-EGFP reporter virus. Additional information was observed using cell culture derived HCV and CD81 cross-linking as a model for virus cell interactions.

SR-B1 was identified as a co-receptor for HCV by its ability to bind recombinant HCV E2 (2). SR-B1 is highly expressed on the surface of hepatocytes, where it plays a vital role in binding lipoproteins and promoting cholesterol uptake^38^. We did perform FACS analysis and immunofluoresence of IFI6 expressing cells and found no change in SR-B1 expression as compared to control cells (data not shown), suggesting that IFI6 does not target SR-B1.

HCV entry is dependent on co-receptor complex formation between CD81, a tetraspanin superfamily member, and the tight junction protein CLDN1 on host cell membranes. CD81 is a cell surface tetraspanin that binds to HCV E2, and mediates a post-attachment event in virus entry^1^,^39^. The downstream receptor function of CD81 in HCV entry requires an interaction with CLDN1^40^. CLDN1 is a plasma membrane protein that localizes to tight junctions and to the basolateral surface of hepatocytes^41^. Although CLDN1 does not directly interact with HCV glycoproteins, it contributes to the post-binding steps of HCV entry by interacting with CD81, and facilitating virus internalization^3^. Human occludin has been shown to be required for susceptibility to HCV infection of human hepatoma cells^4^. These results fit a model where the tight junction region is the last to be encountered by the virion prior to internalization. CLDN1 reduces the movement of occludin, which is highly mobile and increases occludin at cell-cell contacts^42^. An amino acid substitution in CLDN1 exclusively localizes the protein to the cytosol and abolishes HCVpp entry^43^. Furthermore, inhibitors of protein kinase A (PKA) inhibit the interaction between CD81 and CLDN1, leading to CLDN1 internalization from the cell surface and inhibition of HCV entry^44^.

Previous studies indicated a significant co-localization of CLDN1 and CD81 after cross-linking of CD81 with a specific antibody^7^,^11^,^30^. Our results showed that IFI6 expression results in a significant decrease in CD81 and CLDN1 interactions in following HCVcc inoculation or CD81 cross-linked that would inhibit HCV infection at the point of virus entry. Earlier studies of other flaviviruses identified an IFI6 associated interference of infection at a point well beyond entry^23^,^25^. Further, IFI6 mediated inhibition of HCV replicon function has previously been identified^28^. Our data also indicates that IFI6 disrupts HCV infection at a point prior to virus replication, this indicates a potential mechanism for interference by IFI6 that had not been previously identified. These results are reinforced with additional work using a pseudotyped virion on a non-HCV backbone, as interference of infection clearly separated the activity of the HCV glycoproteins from post-entry virus replication.

Epithelial tight junctions form a selective barrier to the diffusion of toxins, allergens, and pathogens from the external environment into the gastrointestinal tract, lung, liver, and kidney^45^. Hydrogen peroxide disrupts tight junctions by a tyrosine kinase-dependent mechanism^46^. The binding of HCV to human hepatoma cells induces EGFR activation which is dependent on the interactions between HCV and CD81. Recently, Diao et al.^27^ found that the binding of HCVcc particles to cells promotes the co-localization of CD81 with EGFR, with activation of EGFR signaling, and that EGFR is required at a step between CD81–CLDN1 engagement and clathrin-mediated endocytosis. EGFR activation could also be induced by antibody mediated cross-linking of CD81. In addition to its role in HCV entry, EGFR signaling might render cells more permissive to viral infection by antagonizing the antiviral response of type I interferons^47^. Thus, the induction of IFI6 in HCV permissive cells may inhibit infection by a number of mechanisms, and inhibition of IFI6 expression during HCV infection may enhance viral persistence and chronicity of infection.

HCV entry requires HRas, a membrane-bound GTPase associated with EGFR signaling, which aids EGFR-mediated HCV entry^9^. HRas associates with CD81 and promotes its lateral diffusion into stable CD81–CLDN1 complexes. Furthermore, the anticancer compound tipifarnib prevents the membrane localization of HRas and markedly inhibits HCV entry. Our results provide evidence that EGFR activation is inhibited in cells expressing IFI6 and that this inhibition is specific to the activation of CD81 during HCV infection, as IFI6 did not inhibit phosphorylation of EGFR in response to EGF. Further, downstream activation of Raf-1 by HCV infection was also compromised by IFI6 expression. Interestingly, a clear difference in kinase activation associated with HCV was apparent, as compared to CD81 cross-linking. An additional lesion in ERK signaling was noted to occur only in HCV infected cells expressing IFI6, and not in CD81 antibody activated cells. However, modulation of upstream Raf-1 kinase activation was consistent between CD81 activation and HCVcc exposure, indicating a potential difference in the signaling pathways associated with HCV infection beyond those associated with CD81 mediated signaling. In conclusion, IFI6 modulated HCV infection at the level of virus entry by disrupting EGFR activation and HRas/Raf-1 signaling and downstream CD81-CLDN1 interactions in response to HCV infection or CD81 crosslinking.

## Methods

### Cells, plasmids, and antibodies

Immortalized human hepatocytes (IHH) stably expressing of HCV core protein^48^, Huh7.5 cells were maintained in Dulbecco's modified minimal essential medium supplemented with 10% fetal calf serum at 37°C in a 5% CO_2_ atmosphere. Cells stably transfected with IFI6 or IFI27 plasmid DNA (Huh7.5/IFI6-FLAG or Huh7.5/IFI27-FLAG cells) were pooled and maintained in culture medium containing G418 to a final concentration of 800 μg/ml. Antibodies to tubulin, actin and FLAG, CD81 (JS81, Millipore), Claudin, and phosphorylated Raf, EGFR, and ERK and FLAG were procured.

### Generation of HCVcc

HCV genotype 2a (clone JFH1) or EGFP tagged virus were grown in Huh7.5 cells as previously described^49^,^50^.

### Transient Transfection of ISG expressing cell lines

Huh7.5 cells were seeded onto 35 mm dishes 16 hours prior to transfection with IFI6 or control plasmids using ExtremeGENE HP transfection reagent (Roche). Cells were utilized for infection by HCVcc or JFH1-GFP 24 hours after transfection and used as described. Western blot analysis of clarified lysates, and expression of IFI6 by RT-PCR were carried out using cells collected 48 hours after transfection.

### Quantitative RT-PCR for HCV RNA and IFI6

To measure cell-associated HCV RNA levels, and levels of IFI6, RNA was extracted using TRIZOL, following manufacturer's instruction. HCV amplification was performed as previously described^50^ in a single tube by reverse transcriptase–polymerase chain reaction (RT-PCR) in accordance with the manufacturer's guidelines (Superscript III kit; Promega) using a 7500Fast real-time PCR machine (ABI, Foster City, CA). In all reactions the 18S RNA was included as an internal endogenous control for amplification efficiency and RNA quantification.

### Assay for infectivity of cell culture grown HCV

To quantify virus infectivity in cells expressing ISGs, IFI6 or IFI27, a predetermined titer (moi of ~1) of HCVcc (clone JFH1) were incubated with cells for 5 hours at 37°C. Cells were rinsed twice and incubated at 37°C for 96 hours. Cells were rinsed, lysed, and RNA was isolated using the RNAeasy Miniprep kit (Qiagen). cDNA was generated using a Superscript III kit (Invitrogen), followed by real-time PCR to quantify viral RNA titer^50^. A curve of virus dilutions was used as a comparative control, and 18S RNA was used as an internal control. HCV JFH1-EGFP was utilized in a similar manner. GFP detection was carried out by immunofluorescence 48 h after infection or as indicated^49^.

### Pseudotype virus

HIV derived HCV pseudotypes (HCVpp) were generated by the expression of an unmodified E1-E2 genomic sequence (corresponding to amino acid residues 174 to 746) from genotypes 1a (clone H77) or genotype 2a (clone JFH1)^5^. Briefly, HIV pseudotypes were generated by the cotransfection of a subclone of human embryonic kidney epithelial cells (293FT; Invitrogen) with equal quantities of plasmid DNA (4 μg total/35-mm^2^ dish) expressing the full-length HCV glycoprotein region, or empty vector (negative control) and the envelope-defective pNL4.3.Luc.R^−^E^−^ proviral genome by using ExtremeGENE HP. To minimize artifacts, infectivity is expressed as relative luciferase units (RLU). Target cells were seeded into 24-well plates and incubated with viral supernatants in 3% fetal bovine serum-Dulbecco's modified Eagle's medium containing polybrene (5 μg/ml). Cells were washed and lysed with 50 μl of reporter lysis buffer after 72 hours of incubation. Cell lysates (20 μl) were analyzed for luciferase activity by the addition of substrate in a luminometer.

### Flowcytometry

Cell surface expression of CD81 and CLDN1 co- receptors were quantified by flow cytometry using specific antibodies by a Becton Dickinson flow cytometer. Surface marker expression on gated cells was analyzed using FlowJo (Tree Star) and CellQuest (BD Immunocytometry Systems) softwares. The Statistica program was used for analyses of variations.

### Immunofluoresence

Huh7.5 cells or IFI6 expressing Huh7.5 cells were incubated with anti-CD81 MAb JS-81 (10 μg/ml) for 1 hour at 37°C. Cells were fixed in ethanol: acetic acid (95:5), and permeabilized with 0.1% Triton X-100 before staining. Cells were incubated with the indicated primary antibodies for 1 hour at room temperature, washed, incubated with fluorochrome-conjugated secondary antibodies for 1 hour at room temperature, washed, and stained nucleus with DAPI. To correlate this effect with infection by HCVcc, cells were adsorbed with 0.5 moi cell culture derived HCV(JFH1 clone) for 2 hours at 4°C, followed by an additional incubation at 37°C for 1 h. Cells were rinsed and fixed before confocal microscopy (Olympus FV1000). Positive co-localization was measured by Pearsons coefficient, and a co-localization coefficient was established using Olympus Fluoview software.

For analysis of IFI6 subcellular localization, cells were treated with MitoTracker Red (Invitrogen) for 30 minutes prior to fixation. Next, cells were fixed with ethanol: acetic acid; followed by permeabilization with 0.1% Triton X100. Cells were rinsed and incubated with FLAG specific antibody, followed by incubation with a secondary antibody conjugated to AlexaFluor 488. Nuclei were stained with DAPI and cells were examined using a fluorescence microscope (Leica, Model DMI4000B).

### Western blot analysis

Control and IFI6 expressing Huh7.5 cells were incubated with antibody to CD81 (JS-81: 2 μg/ml) for 1 hour at 37°C or with EGF (1 μg/ml) for 15 min or 60 min. Huh7.5 cells or IFI6 expressing Huh7.5 cells were incubated with HCVcc (clone JFH1- moi~3) for 1 or 3 hours. Cell lysates were prepared in the presence of protease inhibitors, clarified by centrifugation, and stored in sample reducing buffer at −70°C prior to use. For protein expression upon HCVcc exposure, IFI6 expressing and control Huh7.5 cells were infected with cell culture grown HCV (clone JFH1) at a m.o.i of 3 for the indicated time points. Cell layers were rinsed with PBS and lysed with SDS-PAGE reducing buffer. Quantification of protein levels were performed using ImageJ software from three separate experiments and relative levels in folds are indicated at the bottom of each panel.

## Author Contributions

Conceived and designed the experiments: R.R., R.B.R. and K.M. Performed the experiments and analyzed data: K.M., Y.C.K., R.R., C.H.H., S.L. and R.B.R. Wrote the paper: R.R., R.B.R., C.H.H. Supervised research: R.R. and R.B.R.

## Supplementary Material

Supplementary Information Gel images

Supplementary Information Supplementary Information
