# Supplementary material for: Interferon-α inducible protein 6 impairs EGFR activation by CD81 and inhibits hepatitis C virus infection
Source: Sci Rep. 2015 Mar 11;5:9012. doi: 10.1038/srep09012 (PMC4355636; doi:10.1038/srep09012)

# INTERFERON-α INDUCIBLE PROTEIN 6 IMPAIRS EGFR ACTIVATION BY CD81 AND INHIBITS HEPATITIS C VIRUS INFECTION

Keith Meyer[1], Young-Chan Kwon[1], Shuanghu Liu[2], Curt H. Hagedorn[3], Ratna B. Ray[4], and Ranjit Ray [1, 5*]

Departments of Internal Medicine[1] and Molecular Microbiology & Immunology, Saint Louis University[5], Department of Medicinal Chemistry, College of Pharmacy, University of Utah[2], Central Arkansas Veterans Healthcare System and University of Arkansas for Medical Sciences[3], and Department of Pathology, Saint Louis University[4]

Whole gel from Figure 2A

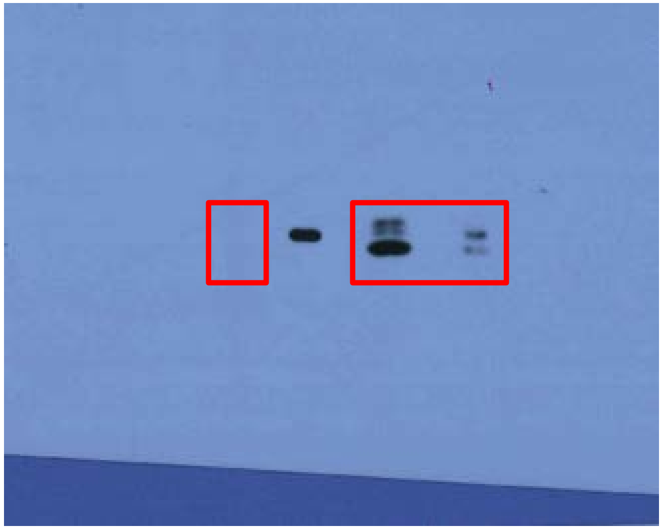

NS3

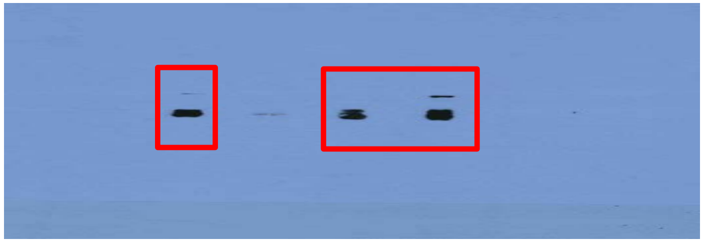

actin

Whole gel from Figure 5A

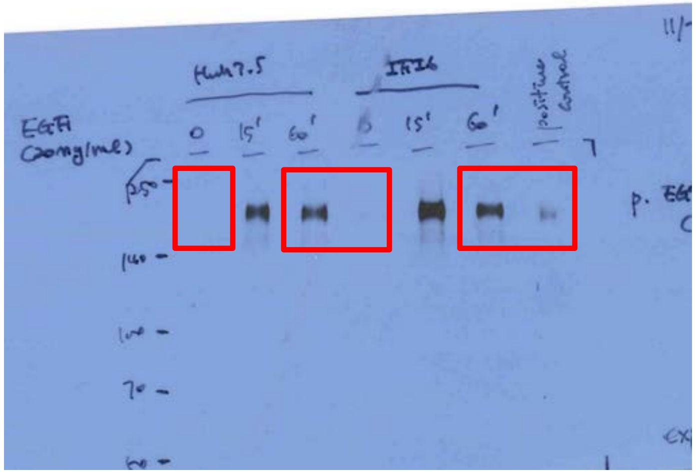

pEGFR Y1068

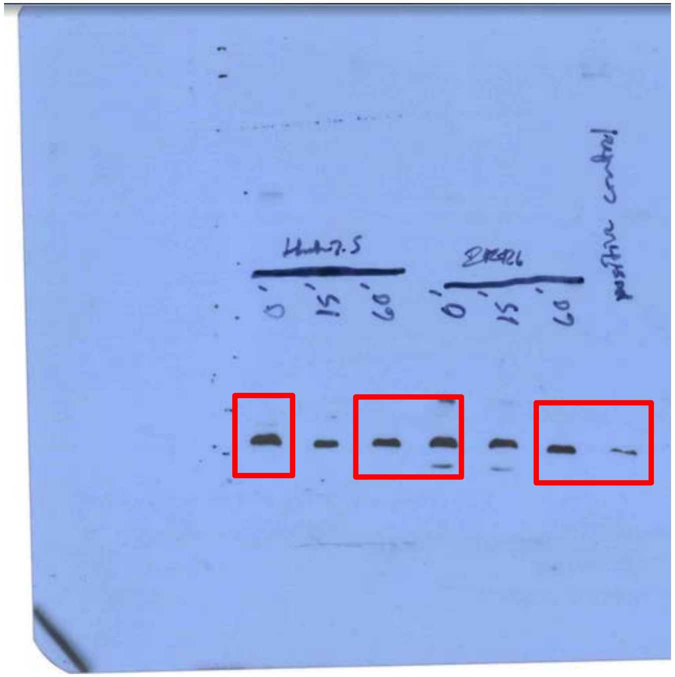

actin

Whole gel from Figure 5B

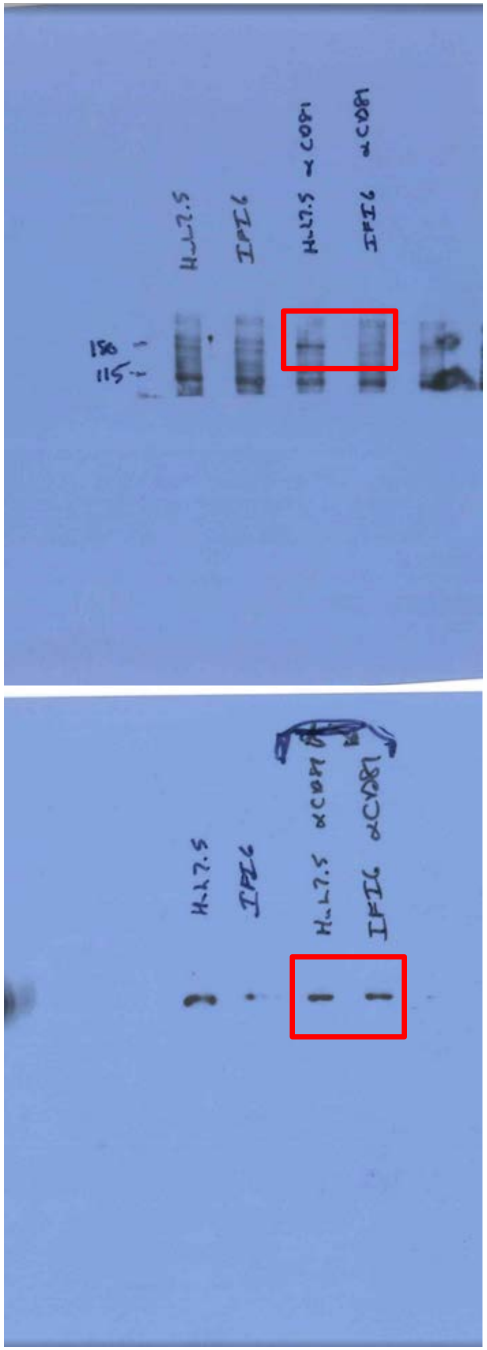

pEGFR Y1068

actin

Whole gel from Figure 6A

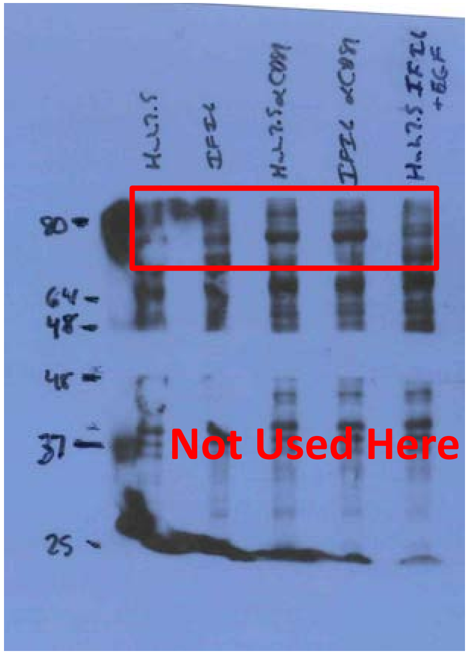


pRaf1 S259

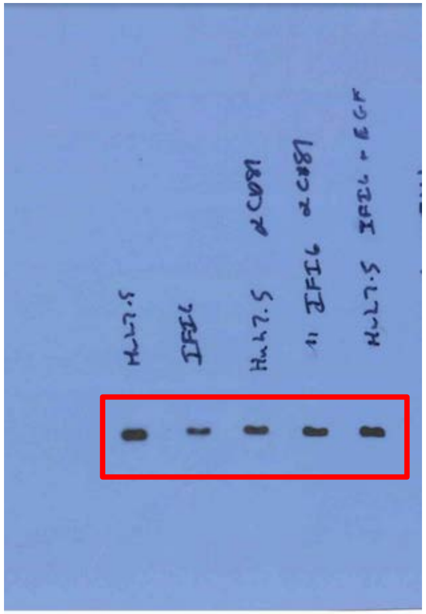

actin

Whole gel from Figure 6B

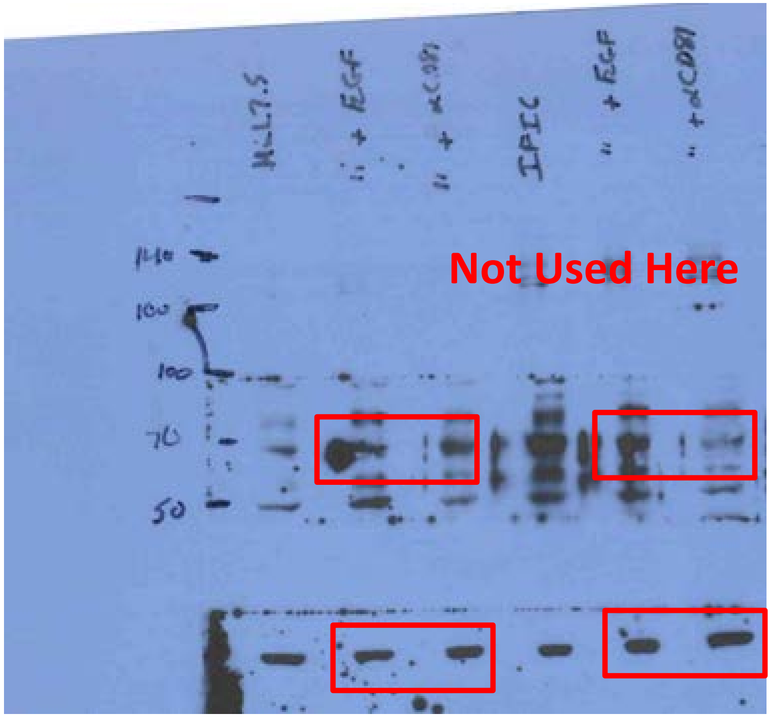


pRaf1 S338

actin

Whole gel from Figure 7A

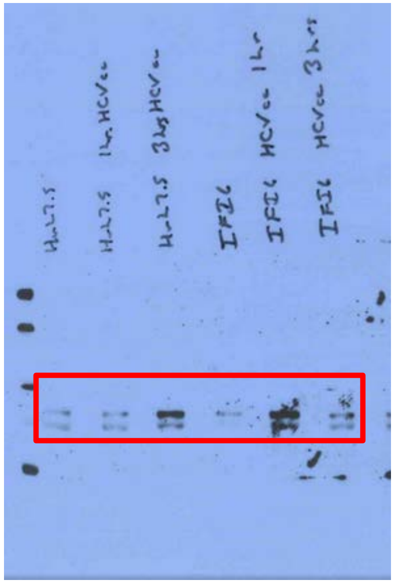

pRaf1 (S259)

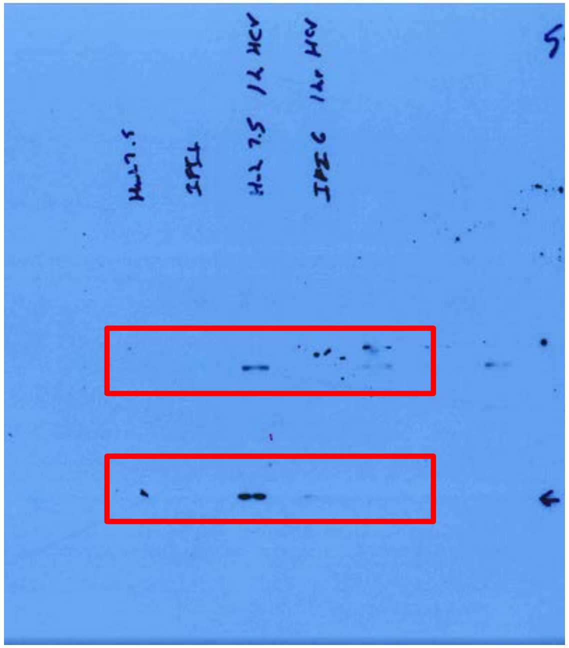

pRaf S338

pERK T202/Y204

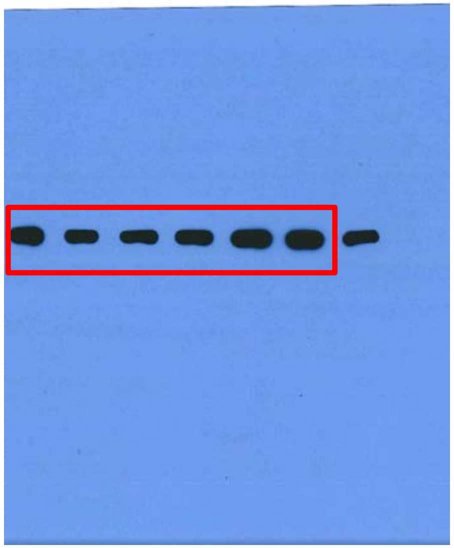

actin

Whole gel from Figure 7B

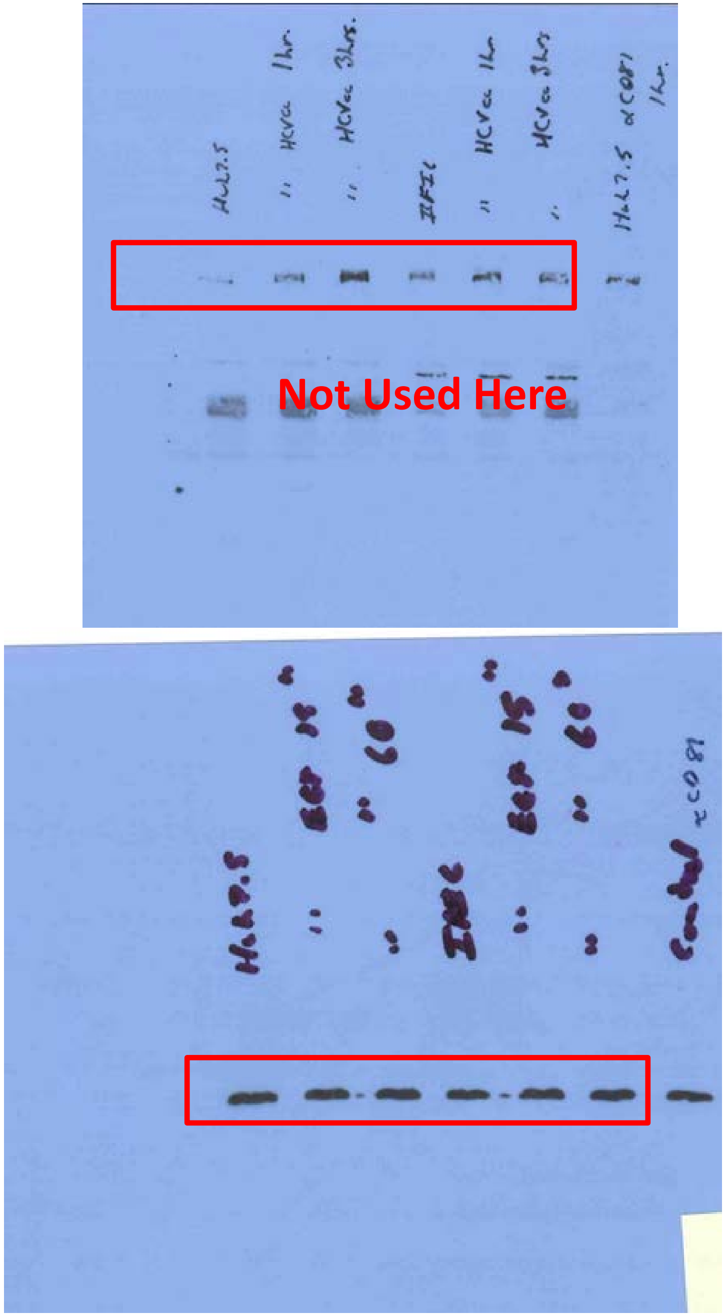


pEGFR Y1068

actin

# Supplemental Figure 1

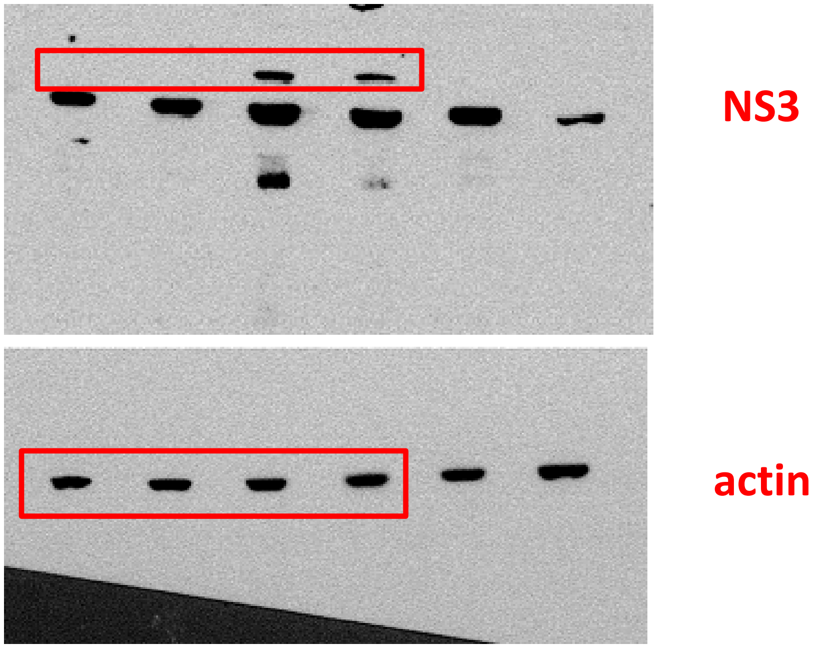

Supplement: Supplementary Information — Gel images [file srep09012-s1.pdf]
