# Supplementary material for: Interferon-α inducible protein 6 impairs EGFR activation by CD81 and inhibits hepatitis C virus infection
Source: Sci Rep. 2015 Mar 11;5:9012. doi: 10.1038/srep09012 (PMC4355636; doi:10.1038/srep09012)
Supplement: Supplementary Information [file srep09012-s2.pdf]

INTERFERON-α INDUCIBLE PROTEIN 6 IMPAIRS EGFR ACTIVATION BY CD81 AND INHIBITS HEPATITIS C VIRUS INFECTION

Keith Meyer[1], Young-Chan Kwon[1], Shuanghu Liu[2], Curt H. Hagedorn[3], Ratna B. Ray[4], and Ranjit Ray [1, 5*]

Departments of Internal Medicine[1] and Molecular Microbiology & Immunology, Saint Louis University[5], Department of Medicinal Chemistry, College of Pharmacy, University of Utah[2], Central Arkansas Veterans Healthcare System and University of Arkansas for Medical Sciences[3], and Department of Pathology, Saint Louis University[4]

Figure S1: Effect of IFI6 on HCV replicon expression. Expression of IFI6 led to a modest reduction in relative replicon RNA levels, and standard deviations are shown as error bars (panel A). NS3 specific protein expression in Rep2a harboring cells is shown. Note that cropped gel images are used in this figure and the gels were run under the same experimental conditions (panel B).

A

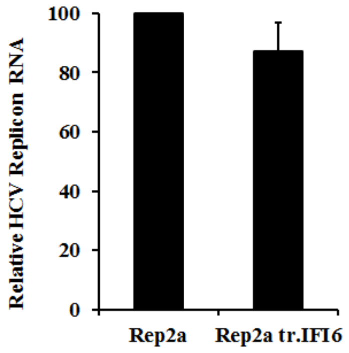
Relative HCV Replicon RNA
100
80
60
40
20
0
Rep2a
Rep2a tr.IFI6

B

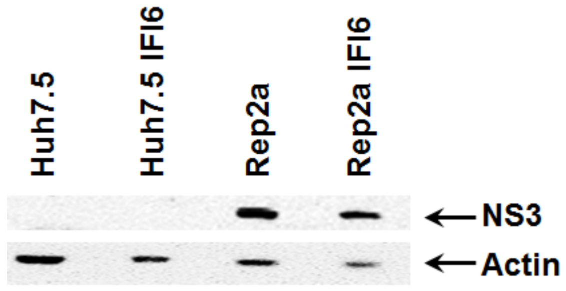
Huh7.5
Huh7.5 IFI6
Rep2a
Rep2a IFI6
NS3
Actin
